# Spatiotemporal dynamics of nanowire growth in a microfluidic reactor

**DOI:** 10.1038/s41378-021-00308-4

**Published:** 2021-10-11

**Authors:** Mazen Erfan, Martine Gnambodoe-Capochichi, Yasser M. Sabry, Diaa Khalil, Yamin Leprince-Wang, Tarik Bourouina

**Affiliations:** 1 ESYCOM, CNRS UMR 9007, Université Gustave Eiffel, ESIEE Paris, Noisy-le-Grand, France; 2 Ain Shams University, Faculty of Engineering, ECE Department, Cairo, Egypt

**Keywords:** Nanowires, Micro-optics, Chemistry

## Abstract

Co-integration of nanomaterials into microdevices poses several technological challenges and presents numerous scientific opportunities that have been addressed in this paper by integrating zinc oxide nanowires (ZnO-NWs) into a microfluidic chamber. In addition to the applications of these combined materials, this work focuses on the study of the growth dynamics and uniformity of nanomaterials in a tiny microfluidic reactor environment. A unique experimental platform was built through the integration of a noninvasive optical characterization technique with the microfluidic reactor. This platform allowed the unprecedented demonstration of time-resolved and spatially resolved monitoring of the in situ growth of NWs, in which the chemicals were continuously fed into the microfluidic reactor. The platform was also used to assess the uniformity of NWs grown quickly in a 10-mm-wide microchamber, which was intentionally chosen to be 20 times wider than those used in previous attempts because it can accommodate applications requiring a large surface of interaction while still taking advantage of submillimeter height. Further observations included the effects of varying the flow rate on the NW diameter and length in addition to a synergetic effect of continuous renewal of the growth solution and the confined environment of the chemical reaction.

## Introduction

The integration of nanomaterials into microscale devices opens an avenue of new opportunities^1–7^ that take advantage of the multiple virtues of these devices, both at the nanoscale and microscale^8,9^. The wide spectrum of available nanomaterials^10,11^ offers a diverse collection of intrinsic physical and chemical properties, which are often enhanced owing to their large specific area^12–16^. In addition, microscale chips benefit from existing infrastructures and technologies and have been shown to enable large volume production at a low unit cost. Consequently, microelectronics, optoelectronics, microelectromechanical systems, and microfluidics appear to be excellent platforms for hosting such nanomaterials toward the emergence of novel functional devices. Operation at the microscale takes additional advantage of favorable scaling laws;^17,18^ in particular, it makes use of the faster physical and chemical phenomena.

Among the different options for nanomaterials, zinc oxide nanowires (ZnO-NWs) have been selected for ease of growth using soft chemistry. They also have numerous interesting properties, which also makes them a good candidate for novel applications. Obtaining well-controlled, high aspect ratios and good-quality nanowire arrays are essential. Several approaches have been utilized to synthesize high-quality ZnO-NWs, such as physical vapor deposition (PVD)^19,20^ and chemical vapor deposition (CVD)^21^. However, many of these techniques are complex and require expensive equipment; in contrast, solution-phase synthesis is an easy and inexpensive way to produce large areas of high-quality ZnO-NWs. Different solution-phase synthesis techniques have been used in the literature, such as electrochemical deposition^22,23^, hydrothermal approach^24,25^, and sol-gel^26,27^, among which hydrothermal growth is one of the easiest and lowest-cost methods, allowing the synthesis of high-quality ZnO-NWs over large areas.

At present, microfluidic devices are attracting increased interest over conventionally sized systems owing to their multiple advantages, such as reduced sample volume consumption, faster reaction on the microscale, cost reduction owing to batch processing, and the ability to perform parallel experiments simultaneously. These advantages drive the use of microfluidics in different applications, such as biology, chemistry, and multidisciplinary applications^28–31^. Further integration of functional nanostructures within microfluidic devices can combine the advantages of both the unique properties of nanomaterials and the diverse functionalities of microfluidics. Much effort has been devoted in the literature to integrating nanomaterials into microfluidics systems either through ex situ or in situ synthesis of nanostructures. Some groups have reported the integration of ex situ fabricated nanostructures in microchannels, where presynthesized nanostructures such as carbon nanotubes and indium phosphide nanowires were assembled by different guiding forces^32–35^. However, these attempts faced different challenges including the positioning of the nanostructures, as accurately positioning them was a very challenging process with limited reproducibility, controllability, and yield. Moreover, adhesion to the surface was very weak, harming the mechanical and electrical robustness. Another method was based on the assembly of the substrate surface, which was already decorated with a ZnO-NW array synthesized using a conventional hydrothermal method, to a polydimethylsiloxane (PDMS) cover or glass cover to obtain the functionalized microfluidic chamber^12,36,37^. In those reports, the authors showed water purification efficiency improvement and achieved highly sensitive infectious pathogen detection through the use of a microfluidic environment. In addition, various studies have been devoted to using the microfluidic environment to grow ZnO-NWs in situ within microchannels whose width does not exceed 500 µm^38–40^. A combination of in situ and conventional hydrothermal growth was proposed by Kim et al.^41^ and Mehare et al.^42^ Furthermore, Luo et al.^43^ succeeded in obtaining ZnO nanostructures, not NWs, locally within capillary tubes. A recent review by Hao et al.^44^ summarizes the different microfluidic approaches for the synthesis of various ZnO micro/nanostructures and highlights their potential application in biosensing, biological separation, and molecular catalysis applications through microfluidic chips.

Similar to all chemical reactions, ZnO-NW growth by the hydrothermal method is ruled by the laws of chemistry, and thus, many factors of the growth process were tested in the aforementioned works to study their effect on NW morphology. For instance, Kim et al.^38^ controlled the synthesized NWs by changing the seed layer preparation, synthesis time, and heating locations within microchannels of 10–20 µm height and 100–150 µm width. Furthermore, Guo et al.^40^ tested the impact of changing the polyethyleneimine (PEI) content on the lateral and axial growth of the NWs in 50 µm-high and 500 µm-wide microchannels. They used PEI to inhibit the lateral growth of ZnO and obtain uniform ZnO-NWs, but the effect of the PEI was not as significant as it was on the ZnO-NWs grown in bulk solution. It is worth mentioning that the ZnO-NWs synthesized in the microfluidic channels showed much faster growth in both the lateral and axial dimensions compared to the conventional bulk system. It should be pointed out that characterization is usually performed using scanning electron microscope (SEM) after the NW growth is complete and the microfluidic device is destroyed to enable side-view observation. In contrast, real-time monitoring of NW growth in situ would provide more insights into the characteristics of the NWs grown within the microfluidic reactor. It would also provide the chance to use the microfluidic device in its intended application without the need to destroy it for the characterization of the embedded NWs.

**Fig. 1:**
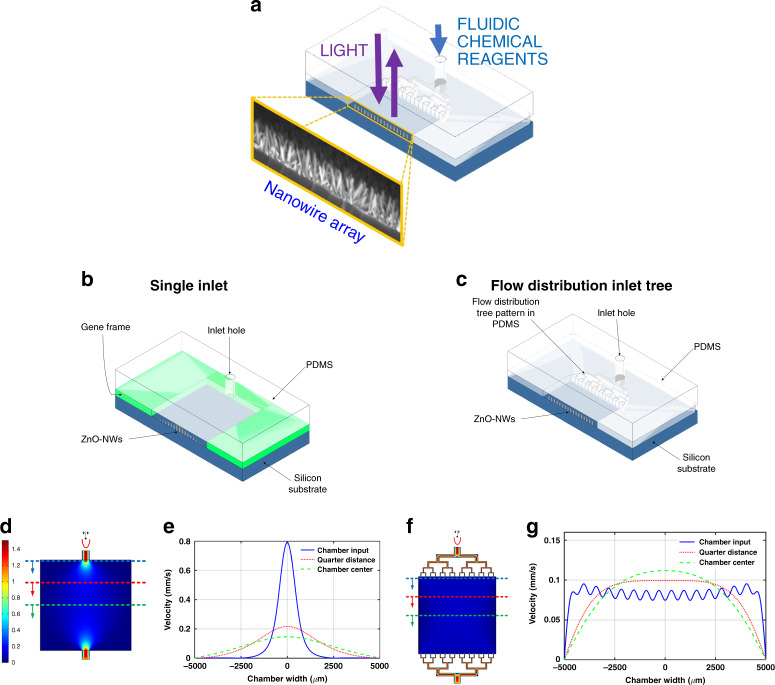
ZnO-NWs integration in microfluidic reactor illustration. **a** Schematic representation of the in situ synthesis of nanowires in a microscale fluidic reactor with noninvasive growth monitoring using light. Illustrative schematics of the two proposed reactors: **b** reactor with a single inlet using a gene frame to form the microfluidic space, **c** reactor with a flow distribution tree molded with soft lithography. COMSOL simulations for a 10 mm × 10 mm reactor with a single inlet in **d** and a four-level biomimetic flow distribution design in **f** showing the flow velocity distributions and the velocity profiles at different positions along with the reactor **e**–**g**

In this work, we report fast and efficient in situ synthesis and real-time monitoring of NWs grown over intentionally wide, centimeter-scale microfluidic reactors that have all the advantages of micrometric-scale reactors, including the out-of-plane dimension, that is, the microreactor height, as shown schematically in Fig. 1a; in addition, real-time monitoring of the growth kinetics is achieved noninvasively using light. The growth is carried out in a dynamic mode involving the continuous flow of the growth solution inside the microreactor. Excellent quality and well-oriented ZnO-NWs are grown uniformly across the whole wide chamber (10-mm-wide) thanks to the designed flow distribution tree based on a biomimetic approach intended to ensure uniform flow. NW arrays are achieved in only minutes rather than in a few hours, which is the time needed for the conventional hydrothermal method under static mode on the macroscopic scale. The impact of different growth parameters, such as flow rate and reaction time, was tested to achieve the best conditions for in situ growth. For the first time, to the best of our knowledge, time-resolved and spatially resolved online monitoring of NWs growth within a microfluidic reactor was implemented noninvasively, hence avoiding cleaving the samples, as in conventional characterization methods such as SEM. This was achieved thanks to the integration of our recently proposed characterization method, spectral-domain attenuated reflectometry (SDAR)^45^, which is proven in this study to be very effective for noncontact, real-time characterization of NWs in situ dynamic growth within a microfluidic reactor. The obtained quantitative results were confirmed using SEM as a reference characterization tool.

## Results and discussion

A hydrothermal in situ method is proposed to synthesize ZnO-NWs on seeded silicon (Si) substrate within a microfluidic chip in dynamic mode instead of the static mode previously reported in the literature^13,24,25,45^. This method has all the advantages of a microfluidic environment. The small volume of the reaction microreactor and the continuous renewal of the chemical solutions provide an additional advantage for the fast growth of ZnO-NWs, which occurs in minutes rather than over a few hours, as is required in the static mode. Two approaches have been tested to produce NWs in situ in wide reactors, as shown in the schematics in Fig. 1b, c and their corresponding fluid flow COMSOL simulations in Fig. 1d–g.

**Fig. 2:**
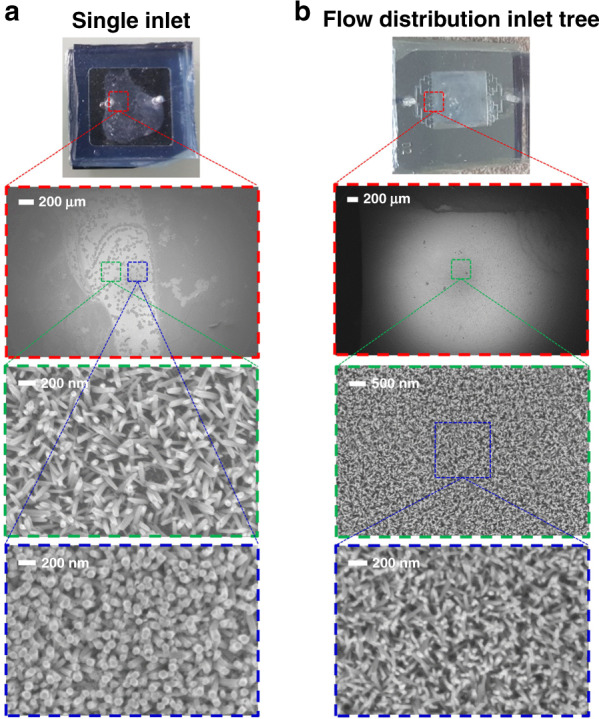
Uniform versus nonuniform in situ growth within a wide surface microfluidic reactor. **a** Photograph and SEM images showing the nonuniformity of the NWs; the top-view image is shown at the top and the different morphologies obtained simultaneously on the same chip are shown in the magnified views below. **b** Photograph and SEM images of the ZnO-NWs grown uniformly in the wide microreactor and the corresponding magnified images to show the quality of the obtained NWs. At the top of each column, the photographs of the microfluidic reactors after finishing the NWs growth show (in light gray) the uniformity of the growth in both cases

First, the basic approach is based on feeding the reactor with a single inlet. NWs with acceptable quality were grown in 16 min and with a 2 mL/min flow rate, as shown in the SEM images in Fig. 2a.

The challenge of this approach is the nonuniform flow of the growth solution in the wide reactor, which causes nonuniform growth of the NWs. COMSOL Multiphysics was used to simulate the fluid flow in the microreactor with 10 mm×10 mm dimensions and a single inlet of 0.8 mm width. Much nonuniformity in the flow can be seen from the surface velocity color map and the cross-sectional curves at specific positions in the reactor, as shown in Fig. 1e; this observation was expected because the fluid flow in microfluidics is mostly laminar with a parabolic velocity profile. This trend holds true when a small inlet supplies the fluid to a wide reactor, where the fluid velocity is generally at its highest in the middle of the microreactor and at its lowest at the edge. The nonuniformity of the NW growth can be easily noticed from the chip photography image in Fig. 2a and from the wide top-view SEM image where some positions have no NWs at all as in the edge and even in the middle area where the difference could be noticed from the difference in the color (the light gray areas are the grown NWs, while dark gray areas are the silicon chip surface). This inhomogeneity can be seen from the different morphologies obtained on the same chip, as shown in the SEM images at different zooms in Fig. 2a.

To overcome this challenge, a well-designed flow distribution network was introduced to achieve improved uniformity of the flow within the microfluidic reactor, as shown by the simulation in Fig. 1f. In this case, the reactor was fabricated by soft lithography to draw the required microchannel design patterns in the PDMS, as will be described in detail in the Materials and Methods section. As shown from the simulation in Fig. 1g, better flow uniformity is achieved using the biomimetic flow distribution tree (Supplementary information 1) than with the single inlet reactor, as shown in Fig. 1e^46,47^. Uniform ZnO-NWs was achieved over the whole microreactor surface, as shown in the SEM images at different zooms in Fig. 2b and in the additional SEM images at different positions, as shown in Supplementary Information 2, where the NWs grew in 30 min and with a flow rate of 1 mL/min. The ZnO-NWs were grown vertically in a random forest fashion with a maximum tilting angle of 10° with respect to the direction normal to the surface, as shown in the acquired SEM images. This growth is similar to those of the NWs grown at the macroscale, where preferential growth occurs along the *c* axis of the ZnO Wurtzite lattice, as already confirmed by X-ray diffraction (XRD) patterns, in which the largest XRD peak corresponds to the [002] crystallographic plane of ZnO^45^. Uniform growth of NWs was achieved over a 10-mm-wide microreactor, as shown in the figure inset, which is 20× larger than the previously reported in situ growth in microchannels, which was limited to widths below 500 µm^38–40^. Such a wide microreactor is important in some applications, such as efficient water purification using a photocatalytic nanomaterial layer, which requires a large surface for polluted water to interact with the photocatalyst^12,47^.

**Fig. 3:**
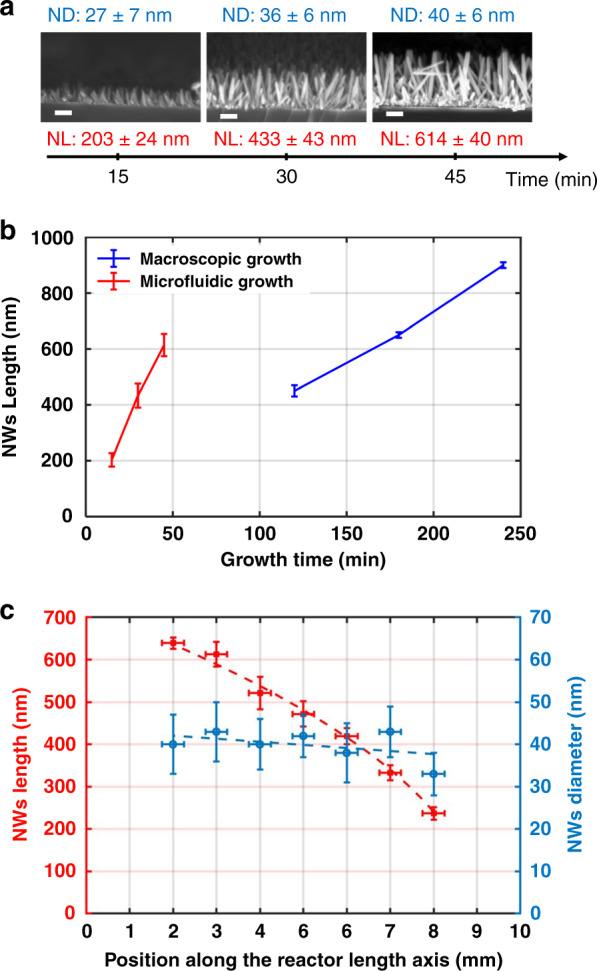
Growth time effect on the NWs in situ growth. **a** Summary of the ZnO-NW length (NL) and diameter (ND) measured from the cross-sectional side-view SEM images showing the effect of the growth time on NW growth when the flow rate was fixed to 1 mL/min. The scale bar represents 200 nm. **b** Comparison between the ZnO-NW length (and growth rate—given by the slopes) achieved through microfluidic and conventional methods at the macroscopic scale. **c** NW length gradient across the reactor length in the flow direction; the NW diameters are slightly varied

One of the parameters that control the NW characteristics is the growth time. Increasing the growth time is expected to increase the NW size, following a similar trend (although it is faster) as conventional macroscale growth^13,48^. The effect of the growth time is tested on chips with a biomimetic flow distribution tree to ensure uniform flow while the flow rate is fixed at 1 mL/min and the growth solution concentration is constant. Cross-sectional side-view SEM images of the NWs grown for 15, 30, and 45 min are shown in Fig. 3a along with a summary of the achieved NWs length and diameter measured from these SEM images. Quite long NWs could be achieved in situ in <1 h of growth, which is not achievable with macroscale conventional hydrothermal growth^48^. This growth was achieved because the height of the microfluidic environment was confined, which, along with continuous chemical refreshment, accelerated the chemical reaction. A comparison between the achieved NWs lengths in the in situ microfluidic environments and at the macroscale is presented graphically in Fig. 3b; the same NW length can be achieved four times faster in this specific microfluidic environment. For instance, NWs of ~600 nm is achievable within 45 min in a microfluidic system, though their formation requires almost 3 h on the macroscopic scale.

It is worth noting that all the SEM images presented in Fig. 3a and the corresponding NW lengths shown in Fig. 3b were acquired at the center of the reactor for the sake of equivalent comparison. As an interesting observation, the NW size decreased along the direction of the flow, as shown in Fig. 3c. This observation could be owing to the reduction of the solution concentration across the reactor length because the nearest position to the inlet receives the fresh concentrated solution, which leads to longer NWs; in addition, the active species concentration is reduced because it is used in the chemical reaction, leading to smaller NWs further away from the inlet^38,49^.

**Fig. 4:**
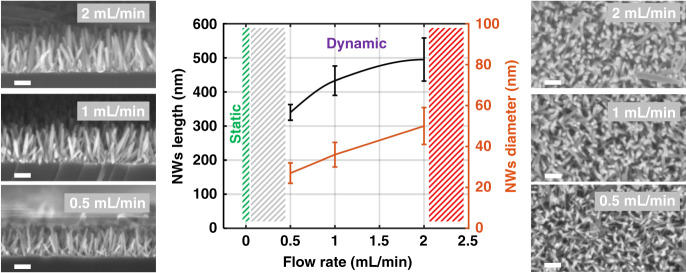
Flow rate effect on the NWs in situ growth. Summary of the NW length and diameter versus flow rate with the top- and cross-sectional side-view SEM images showing the effect of the flow rate on NW growth when the growth time was fixed to 30 min. The scale bar represents 200 nm

Another important parameter that could affect the NW characteristics is the flow rate, which affects the refreshment of the growth solution as growth occurs in dynamic mode, which involves the continuous flow of the growth solution inside the microreactor. The effect of the flow rate was examined on chips similar to those used for the growth time effect experiment, while the growth time was fixed here to 30 min and with constant growth solution concentration. Top- and cross-sectional side-view SEM images of the NWs grown with flow rates of 0.5, 1, and 2 mL/min are shown in Fig. 4. It is worth mentioning that no NWs were observed with slower flow rates (such as 0.25 mL/min), as more growth time was needed to have significant growth of the NWs at such low flow rates, which is understandable since very small NWs usually grow in static mode (a flow rate of zero) in 30 min^48^. One other possible reason for the absence of NW growth at low flow rates could be the mixing of the inhomogeneous growth solution in the reactor; however, in our case, the solution was well mixed in glass flasks before injection into the reactor, which did not include mixers but only a reaction chamber. Nevertheless, it will be quite interesting to perform further investigations with a small flow rate but with a longer growth time to assess the effect of solution refreshment versus the confined scale of the reaction. In contrast, increasing the flow rate above 2 mL/min is not preferable, as this increases the pressure significantly on the microfluidic reactor, which could lead to PDMS-substrate interface detachment. It also increases the fluid surface velocity, which is not suitable for growing NWs of good quality. A summary of the achieved NWs length and diameter measured from the SEM images is presented graphically in the middle of Fig. 4. It is interesting to note that increasing the flow rate has a greater effect on increasing the diameter rather than on increasing the length, as the NWs can be imagined as growing layer-by-layer, and the higher flow rate involves a spread on the NW diameter. It is worth mentioning that in all the previous characterizations, all the SEM images were acquired after detaching the PDMS from the substrate and destroying the reactor, which was necessary to perform the SEM measurements.

To characterize the NWs grown within the reactor without destruction and to be able to use them later for their intended application, a noninvasive technique is needed. Contactless and real-time monitoring of the in situ growth of the NWs within microfluidics reactors was achievable through the integration of the SDAR technique^45^ with the in situ growth setup. The whole growth and monitoring setup are described in detail in the Materials and Methods section, which details a newly added Y-fiber cable reflection probe that perpendicularly illuminates and collects the reflected light remotely through the PDMS that covers the microreactor. This Y-cable delivers light from the light source and collects the reflected light to the UV-vis-NIR spectrometer to enable the desired real-time monitoring. All the monitoring processes were carried out with a fixed flow rate at 1 mL/min, and the growth time was 30 min. It is important to note that the monitored NWs were at the center of the microchamber midway between the inlet and outlet. In addition, the reported values of the NWs length consist of a local average over a 1 mm-diameter area, corresponding to the size (at the sample surface) of the light spot originating from the optical fiber used for these measurements.

**Fig. 5:**
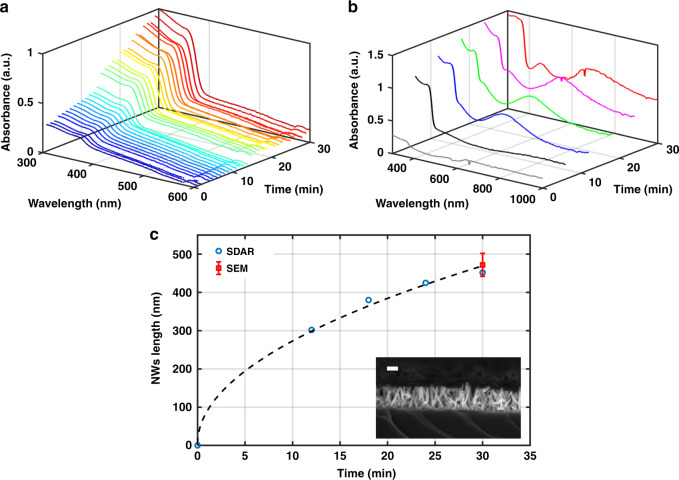
Noninvasive real-time monitoring of the NWs in situ growth. **a** UV-vis spectra measured every 1 min for 30 min showing the ZnO-NW bandgap absorption evolution. **b** Online monitoring of ZnO-NWs growth using UV-vis-NIR spectroscopy, where the NW length was retrieved from the interference pattern in the vis-NIR range. **c** NW lengths were retrieved from the UV-vis-NIR spectra using the SDAR technique at different time points during the growth and the final measured length determined from the SEM image (redpoint) after the completion of growth. In the inset, the side-view SEM image of the monitored ZnO-NWs was taken after the completion of growth; the scale bar represents 200 nm

Monitoring was performed by acquiring a spectrum every 1 min for 30 min without any interruption to the growth solution flow. The evolution of the ZnO bandgap absorption during the growth, shown in Fig. 5a, indicates that this type of absorption starts to increase significantly just after a few minutes owing to the fast growth in the confined environment of the microreactor. The increase in absorbance corresponds to an increase in the ZnO amount. The sharper bandgap cutoff and the increase in the cutoff wavelength correspond to the improvement in the crystallinity of the structure over time^45^. However, no interference pattern was acquired above the cutoff wavelength, which prevented the ability to retrieve the NW length from it. This limitation could be due to the flow of the solution liquid or the chemical byproducts, which could lead to scattering of the specularly reflected light responsible for the ripples in the spectra.

Therefore, another approach was used for online monitoring of the NW length. Two syringes were used: one contained the growth solution, and the other was filled with air. After 5 min of growth solution flow in the reactor, the input tube was connected to the air syringe and air was circulated through the system for 1 min to ensure that it filled the reactor during spectrum acquisition. Thus, online monitoring of the NW length growth was achieved. The series of spectra acquired every 6 min (5 + 1 min) is shown in Fig. 5b, demonstrating the evolution of the interference peaks above the cutoff wavelength. These interferences represent the increase in the NW length. It is noteworthy that the baseline shifts up with time, which could be due to the collection of more scattered (diffuse reflected) light due to the increase in the NW size over time, where the optical setup collects both diffuse and specular reflections. The NW length was then extracted from the interference pattern following a similar procedure discussed in our prior work describing the SDAR method^45^. The process of retrieving the NW thickness uses the interference pattern contrast and the spectral periodicity in the reflection response, which involves an automated best fit of the measured spectra with simple theoretical modeling based on the effective medium approach.

A summary of the lengths retrieved over time is presented in Fig. 5c, and the final NW length (redpoint) was confirmed using an SEM image acquired after growth was complete; this result is presented in the inset of Fig. 5c and shows very good agreement with the monitored length. As shown in the figure, the evolution of the NW length over time shows two growth stages, with a higher growth rate in the first stage; this result is in agreement with similar observations of macroscale growth reported in the literature^25,48^. It is noteworthy that the interference pattern contrast was very small as growth began owing to the small length and density of the NWs, which made it impossible to extract the exact NW length in this particular state. However, in this experiment, we exhibit NWs that were slightly longer than those used in the previous experiment of in situ growth, which tested the effect of the growth time.

## Conclusion

In summary, taking advantage of a microfluidic environment, we reported a time-resolved and spatially resolved dynamics study and online monitoring of the in situ growth of ZnO-NWs within a microfluidic reactor, showing the impact of different growth parameters on NW characteristics. Growth was carried out in dynamic mode under a continuous flow of the growth solution inside the microreactor. The microfluidic reactor consisted of a silicon base containing the ZnO seed layer, which was bonded to a PDMS piece containing the microreactor and microchannels. Well-oriented ZnO-NWs of excellent quality were grown uniformly across the whole microreactor through the uniform flow distribution resulting from the designed biomimetic tree architecture that uniformly fed the growth solution into the microreactor reactor. The ZnO-NWs were grown successfully in a microreactor with 10 mm lateral dimensions, which is rather large in comparison with the maximum width of the microchannels considered thus far in the literature, which was only 500 µm. Owing to the continuous renewal of the growth solution and the confined environment of the chemical reaction, growth is achieved in the microfluidics system at a rate that was four times faster than macroscopic growth. ZnO-NW arrays were achieved in tens of minutes rather than hours, which are needed for the conventional hydrothermal method under static mode. Varying the flow rate had a stronger influence on the NW diameter than on the NW length. For the first time, to the best of our knowledge, we present online monitoring of NW growth within a microfluidic reactor by implementing a noninvasive, remote, and real-time optical characterization method integrated with a growth microreactor. The obtained quantitative results were confirmed by SEM with excellent agreement. These achievements open the door for producing and studying even faster chemical reactions such as NW growth or water photocatalytic purification within microfluidic reactors while simultaneously performing the corresponding real-time monitoring, which enables more comprehensive studies of chemical kinetics on the microscale.

## Materials and methods

In this section, our proposal for in situ growth of ZnO-NWs within wide microreactors is presented. Furthermore, real-time monitoring of the in situ growth of ZnO-NWs in dynamic mode is demonstrated. This demonstration was made possible by implementing our characterization technique, SDAR, which is described in a previous report^45^.

### Growth and monitoring experimental setup

**Fig. 6:**
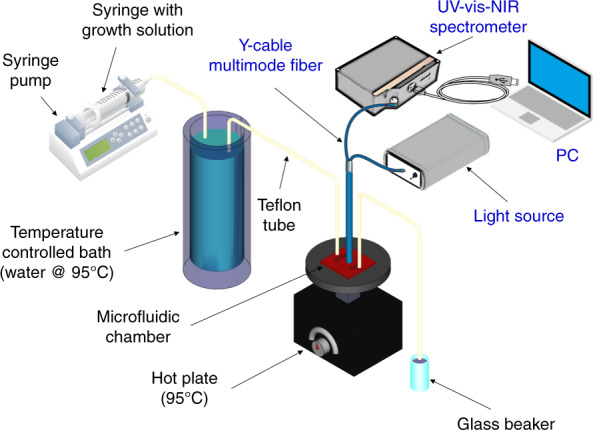
Growth and monitoring setup illustration. The in situ growth of ZnO-NWs and real-time monitoring setup schematic

The growth setup is shown schematically in Fig. 6. The growth setup included a syringe pump (PHD ULTRA, Harvard Apparatus) to precisely control the flow rate and chemically inert Teflon tubes, which were used to carry the growth solution to the reactor and remove the byproducts. The input tube was immersed in hot water at 95°C to heat the growth solution prior to the chemical reaction and to maintain the reaction temperature of the whole microreactor, which was heated on a hot plate at 95°C. The silicon base acted as a good thermal conductor where uniform contact was achieved through good fixation of the chip surface onto the plate, ensuring uniform temperature distribution. The real-time optical monitoring setup was composed of Y-cable multimode fiber (premium-grade bifurcated fibers, Ocean Optics), where the common end tip was positioned just above the PDMS to illuminate the sample perpendicularly and collect the reflected light and deliver it to the UV-vis-NIR spectrometer (MAYA 2000 Pro, Ocean Optics), as shown as part of the growth setup presented in Fig. 6. The first port of the Y-cable carried light from the wideband source (DH-mini, Ocean Optics).

### Nanowire growth

Before growth, a Si substrate was washed with a surfactant solution, DI water in an ultrasonic bath, and a plasma cleaner. After the cleaning process, a buffer solution composed by mixing 1 g of zinc acetate dihydrate (Zn(CH_3_COO)_2_∙2H_2_O, > 98%, Sigma Aldrich) with 10 g of polyvinyl alcohol (PVA, >99%, Aldrich Chemistry) in 100 mL of DI water (18 MΩ cm resistivity) was spin-coated on a bare Si chip. The volume of the spin-coated seed layer was 200 µl cm^−2^ based on the surface area of the sample. The speed of the spin coating was 3000 rpm with an acceleration of 3000 rpm min^−1^ for 1 min. Then, the seeded substrate was subjected to calcination at 500°C for 3 h to remove PVA and form ZnO seeds, which acted as nucleation centers to promote the homogeneous growth of ZnO-NWs in the next step. The second step of the hydrothermal synthesis method consisted of ZnO nanowire growth by using the experimental setup illustrated schematically in Fig. 6. The growth solution was prepared by mixing aqueous solutions of 25 mM zinc nitrate hexahydrate (Zn(NO_3_)_2_∙6H_2_O, 98%, Acros Organics) and 25 mM hexamethylenetetramine (≥99%, Merck KGaA). Then, a syringe was filled with the well-mixed growth solution to start the growth process as described in the previous paragraph and shown schematically in Fig. 6. It is worth noting that the growth solution concentration influenced the growth process; however, the concentrations were fixed in all of the experiments.

### Online monitoring of nanowire growth

Along with the in situ growth, online monitoring was performed using the noninvasive SDAR technique without interruption during growth. The Y-cable multimode fiber common end tip was positioned just above the PDMS to illuminate the sample perpendicularly and collect the reflected light and deliver it to the UV-vis-NIR spectrometer, as shown as part of the growth setup presented in Fig. 6. The first port of the Y-cable carried light from the wideband source. The measured reflectance spectra were fitted by considering the NWs as thin films with an effective refractive index dependent on the density ratio of ZnO to air^50^. The fitting was performed by optical spectrum simulation using the transfer matrix method applied to a multilayer stack and trying to reach the best fitting by minimizing the mean squared difference between simulated and measured spectra (SCOUT, Wolfgang Theiss).

### Microfluidic chip fabrication

Two approaches to fabricating the microfluidic reactor were tested to create ZnO-NWs in situ in wide reactors. The first approach used a gene frame to form the microfluidic reactor, and the other involved molding the PDMS using soft lithography^51,52^. To close the microfluidic chip from the top, in the first approach, a gene frame was used on the side edges of the chip to attach the silicon chip to a flat PDMS layer, which had two holes for inlet/outlet tubing, as shown in Fig. 1b. As an initial trial to synthesize ZnO-NWs in situ in a wide microreactor, a double-sided sticky gene frame was used to form the microfluidic space for the flow of the growth solution. The gene frame height was 250 µm, and its internal reactor dimension was 15 mm × 16 mm. The sticky gene frame was used just on the substrate edges to form the microfluidic space and capped from the top by a (~5 mm) thick flat PDMS layer. This PDMS layer was punched using a biopsy puncher (Razor-Sharp Stainless-Steel Biopsy Punch, Darwin Microfluidics) with a 1.25 mm outer diameter (OD) to have inlet and outlet holes. Chemically inert Teflon tubes (PTFE Tubing, Darwin Microfluidics) with 1.58 mm OD and 0.79 mm inner diameter (ID) were used to carry the growth solution liquids and were connected directly to the PDMS with good sticking and good hermeticity, as the tube OD was larger than the hole diameter in the PDMS. To ensure that there was no leakage from the reactor, the gene frame edges were covered with an additional small amount of uncured PDMS and then cured in the oven. PDMS was prepared by mixing PDMS liquid and curing agent at a ratio of 10:1 to obtain flexible PDMS with good stiffness (SYLGARD 184, Dow Corning). Finally, it is important to recall that the reactor was assembled on the substrate that was already coated with a seed layer.

The first approach suffered from a nonuniform flow of the growth solution in the wide reactor, causing nonuniform growth of the NWs, as shown in Fig. 2a. To overcome this issue, a flow distribution network was designed in a second approach to achieving much better uniformity, as illustrated in Fig. 1c. The dimensions of the tree are summarized in Table S1; the dimensions were optimized (Supplementary Information 1) to achieve uniform flow. In this second approach, soft lithography was used to draw the required microchannel design patterns of the flow distribution tree. The reactor consisted of two parts: a silicon substrate covered with a ZnO seed layer and a PDMS part containing microchannels. Then, both parts were assembled using oxygen (O_2_) plasma. The silicon substrate was covered with a thin seed layer of ZnO by spin coating, as previously discussed, but the main difference here was the whole surface of the silicon was not covered with the seed layer so it could further bond to the PDMS. We used Teflon tape on the side edges of the silicon substrate before spin coating. This method was better because it enabled us to avoid using hydrochloric acid (HCl) acid to remove the seed layer from the substrate edges, as in some cases, it is adsorbed by the seed layer edges and spread by capillary action, hence preventing the growth of the ZnO-NWs, which cannot grow in acidic medium^48^. Afterward, an annealing step was performed at 500°C for 3 h after the spin coating was complete and the Teflon tape was removed. The microstructured PDMS fabrication steps and the reactor assembly can be summarized in the following steps. Step 1 is mold fabrication using a SU-8 photoresist containing the “negative” micropatterns corresponding to the desired microchannels; fabrication is performed according to the steps described in the SU-8 datasheet (SU-8 2075, Microchem)^53^. In Step 2, a mixture of liquid PDMS and a crosslinking agent (to cure the PDMS) is poured into the mold and heated for two hours at a high temperature (75°C). Then, in step 3, once the PDMS is hardened, it can be removed from the mold to obtain a “positive” replica of the microchannels on the PDMS piece. In step 4, the inputs and outputs of the microfluidic device are punched with a PDMS puncher to allow the injection of fluids. Finally, the faces of both the PDMS block with microchannels and the silicon substrate are treated with O_2_ plasma for 2 min under a pressure of 300 mTorr, and then both surfaces are pressed together with light pressure to allow the bonding of the PDMS with the substrate to close the microfluidic chip.

## Supplementary information

Supplementary information 1
