# Supplementary material for: Spatiotemporal dynamics of nanowire growth in a microfluidic reactor
Source: Microsyst Nanoeng. 2021 Oct 11;7:77. doi: 10.1038/s41378-021-00308-4 (PMC8505653; doi:10.1038/s41378-021-00308-4)
Supplement: Supplementary file 1 — Supplementary information 1 [file 41378_2021_308_MOESM1_ESM.docx]

Supplementary Information

Spatiotemporal Dynamics of Nanowires Growth in a Microfluidic Reactor

Mazen Erfan ^a^, Martine Gnambodoe-Capochichi ^a^, Yasser M. Sabry ^b^, Diaa Khalil ^b^, Yamin Leprince-Wang ^a^, Tarik Bourouina ^a^*

^a^ ESYCOM, Univ Gustave Eiffel, CNRS, CNAM, ESIEE Paris, F-77454 Marne-la-Vallée, France.

^b^ Faculty of Engineering, Ain Shams University, 11517 Cairo, Egypt.

* Corresponding author: Tarik Bourouina, ([tarik.bourouina@esiee.fr](mailto:tarik.bourouina@esiee.fr))

KEYWORDS: Microfluidic reactor, *in-situ* nanomaterials synthesis, hydrothermal growth, real-time optical characterization, zinc-oxide nanowires.

1. Optimized flow distribution network

The biomimetic flow distribution network is inspired from the vascular system. The diameter of each sub-branch can be calculated using Murray’s law:^[1]^

| ${D_{0}}^{3}={2^{n}D_{n}}^{3}$ | **(1)** |
| --- | --- |

where *n* is the channel generation level, *D_0_* is the diameter of mother branch and *D_n_* is the diameter of the n^th^ generation of the channel. The direct application of Murray’s law in microfluidics is not possible since the channels cross-section is usually noncircular with the available fabrication techniques. However, it is possible to extend this law to other geometries since there is direct analogy with the hydraulic diameter of the respective cross-sections. The generalization of Murray’s law to rectangular and trapezoidal channels is discussed in detail by Emerson *et al.*^[1]^ For constant-depth rectangular channels, the bio-mimetic design can be obtained by solving the following relationships:^[1]^

| $\alpha_{n}\left( 1+\alpha_{n} \right)Po\left( {\alpha_{n}}^{*} \right)=2^{n} \alpha_{0}\left( 1+\alpha_{0} \right)Po\left( {\alpha_{0}}^{*} \right)$ | **(2)** |
| --- | --- |
| $Po\left( {\alpha_{n}}^{*} \right)=24 [1-a_{1}\left( {\alpha_{n}}^{*} \right)+a_{2}\left( {\alpha_{n}}^{*} \right)^{2}-a_{3}\left( {\alpha_{n}}^{*} \right)^{3}$  $+a_{4}\left( {\alpha_{n}}^{*} \right)^{4}-a_{5}\left( {\alpha_{n}}^{*} \right)^{5}]$ | **(3)** |

where *α_n_* is the cross-section aspect ratio (*α_n_=d*/*W_n_*), *d* is the channel depth, *W* is the channel width, ${\alpha_{n}}^{*}=\alpha_{n}$ if $\alpha_{n}$< 1 or ${\alpha_{n}}^{*}=1/\alpha_{n}$if $\alpha_{n}$> 1, $Po\left( {\alpha_{n}}^{*} \right)$is the Poiseuille number calculated using equation **(3)** where the coefficients are a_1_ = 1.3553, a_2_ = 1.9467, a_3_ = 1.7012, a_4_ = 0.9564, a_5_ = 0.2537. By solving equation **(2)** with the substitution of equation **(3)** inside, the width of each sub-channel can be calculated. The microreactors used in all the experiments have a 4-branching levels and the sub-channels width and length are summarized in **Table S1**.

**Table S1.** Summary of the simulated flow distribution tree dimensions. The reactor size is 10 mm x 10 mm with 4-level branching network where the channel depth is 50 µm. The sub-channel length is chosen to be 10 times of the sub-channel hydraulic diameter.

|  | **n = 0** | **n = 1** | **n = 2** | **n = 3** | **n = 4** |
| --- | --- | --- | --- | --- | --- |
| **Channel Width [µm]** | 800 | 385 | 192 | 99 | 56 |
| **Channel Length [µm]**  ***(L = 10 D_H_)*** | 941 | 885 | 793 | 664 | 528 |

1. ZnO-NWs Uniformity in the Flow Distribution Tree Reactor

Uniform ZnO-NWs was achieved over the whole microreactor surface thanks to the well-designed flow distribution network which has been introduced for the purpose of achieving much better uniformity of the growth solution flow within the microfluidic reactor. SEM images from different positions is shown in **Figure S1** showing the uniformity of the grown NWs across the reactor where the NWs grown in 30-min and with a flow rate of 1 mL/min.


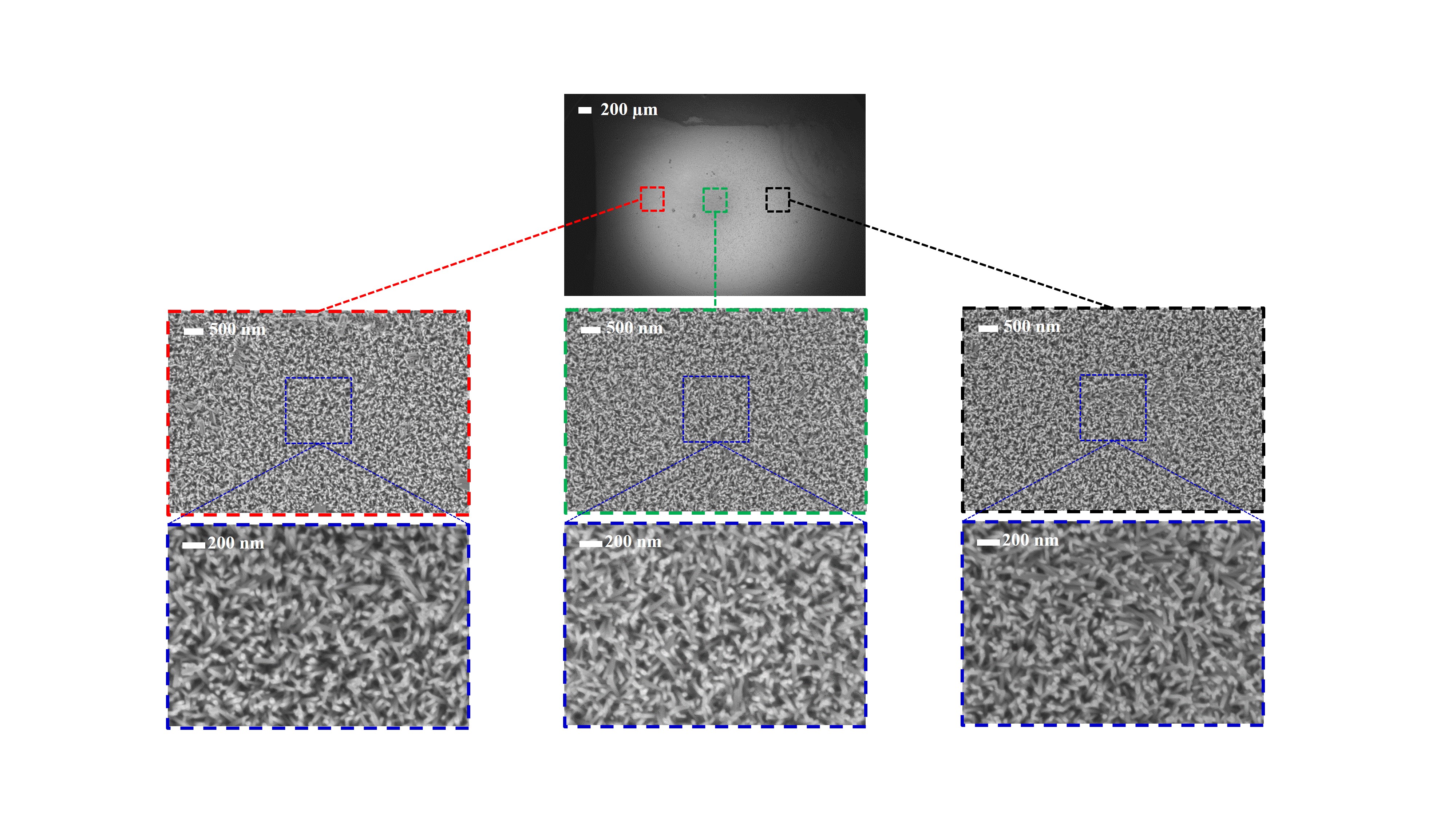


**Figure S1.** SEM images of the grown ZnO-NWs uniformly in the wide microreactor and the corresponding zoomed images to show the quality and uniformity of the obtained NWs at different positions.

1. References
2. Emerson, D. R., Cieślicki, K., Gu, X. & Barber, R. W. Biomimetic design of microfluidic manifolds based on a generalised Murray's law. *Lab chip*. **6**, 447-454 (2006).
